# Between a Rock and a Hard Place – Gender Dysphoria and Comorbid Depression in a Young, Low-Income, Pakistani Transgender Man

**DOI:** 10.7759/cureus.10205

**Published:** 2020-09-02

**Authors:** Usama Irshad, Ali Madeeh Hashmi, Irum Aamer

**Affiliations:** 1 Internal Medicine, King Edward Medical University/Mayo Hospital, Lahore, PAK; 2 Psychiatry, King Edward Medical University/Mayo Hospital, Lahore, PAK

**Keywords:** gender dysphoria, transgender health, lgbtq health, gender identity disorder, pakistan, sex reassignment surgery

## Abstract

Gender dysphoria (GD) describes an incongruence between a person's assigned and expressed gender and the distress associated with it. The management of GD ideally involves a team of healthcare professionals, including psychiatrists, endocrinologists, and surgeons, and may include one or more of the following - psychotherapy, hormone therapy, and gender reassignment surgeries. While Pakistan still has a sizeable population of the traditional 'khwaja siras' or 'hijras,' many young transgender Pakistanis are now adopting global transgender identities and seeking sex-reassignment procedures from a state-run healthcare system, which still heavily discriminates against them. In spite of the passage of a new law protecting the fundamental human rights of transgendered Pakistanis, they continue to be oppressed and deprived of education, employment, and healthcare. This case report describes one such young transman from a low-income Pakistani household, who, owing to the legal red tape, family pressure, religious disapproval, and hefty hospital expenses, is left with only two options - to undergo unsafe, unstandardized procedures at the hands of quacks or live the rest of his life trapped in the wrong body. There is a dire need for a wide-scale awareness campaign to educate and sensitize the general public about transgender rights. Medical students must also be exposed to transgender patients during their medical school training in order to familiarize them with the special medical needs of transgender individuals.

## Introduction

Gender dysphoria(GD) is a new addition to the Diagnostic and Statistical Manual of Mental Disorders (DSM)-5, replacing the previously used term gender identity disorder (GID)*.* It is defined as a marked incongruence between one’s experienced/expressed gender and assigned gender, of at least six months’ duration as manifested by at least two of the following - marked incongruence between one’s experienced/expressed gender and primary and/or secondary sex characteristics, a strong desire to be rid of one’s primary and/or secondary sex characteristics, a strong desire for the primary and/or secondary sex characteristics of the other gender, a strong desire to be of the other gender, a strong desire to be treated as the other gender, and a strong conviction that one has the typical feelings and reactions of the other gender [1].

Pakistan is a country in South Asia with a population exceeding 212.2 million people [2]. The state religion is Sunni Islam. Discrimination and disapproval of the lesbian, gay, bisexual, and transgender (LGBT) community, and the associated social stigma, mostly stem from religious beliefs [3-4].

The term ‘transgender’ has different connotations in Pakistani society [5]. It usually refers to a group of individuals locally known as ‘hijras’ or ‘khwaja siras’ who do not conform to the traditional gender binaries and are widely thought to possess mystical powers. They are invited to sing and dance on occasions like the birth of a male child or a wedding and are supposed to bring good luck. Hijras live in close-knit communities or ‘addas’ formed along the lines of a ‘guru-chela’ relationship dating back to the master-disciple relationship in Sufism and speak a cryptic language called ‘Hijra Farsi’ [5].

In present-day Pakistan, owing to increasing westernization, many young, non-binary, and transgender individuals are breaking away from this traditional system and adopting a global transgender identity. Owing to increasing, albeit reluctant, parental acceptance, many of them opt to stay with their biological parents [6]. In 2018, Pakistan’s Parliament passed the Transgender Persons (Protection of Rights) Act, which, at least on paper, gives every individual the right to have their self-perceived gender identity on all national identification documents and protects the rights of transgender individuals to education, healthcare, and equal employment opportunities [7].

In spite of legal and constitutional protections, transgender individuals continue to face discrimination in accessing medical services, education, housing, transportation, and livelihood opportunities [8-9]. They have been subjected to widespread familial and societal violence, gang-raped, and murdered in cases that have been prominently highlighted in the local and international media [10-11].

In healthcare facilities, transgender individuals face harassment and are often turned away mainly because of the health professional’s ignorance and lack of experience in treating patients who do not conform to the strict gender binaries of the Pakistani society [12]. Across Pakistan, there is no inclusion of transgender healthcare issues in the medical education curriculum. Additionally, doctors are hesitant to perform any gender-reassignment procedures, hormonal or surgical, for fear of religious and legal repercussions. At present, a court order is required to be able to perform a gender reassignment surgery in Pakistan [13].

Gender dysphoric individuals in Pakistan from the upper and middle classes have the financial freedom of traveling to countries like Thailand for sex-reassignment surgeries in safe, private hospital settings, often without their families ever knowing about it [14]. But low-income individuals with limited education and financial freedom do not have this option. Consequently, they are faced with two equally grim possibilities. Many of them, fearing endless legal battles, harassment at healthcare facilities, and hefty hospital expenses, end up taking poorly regulated hormones and becoming victims of dangerous surgical procedures performed by quacks behind closed doors. This leads to medical complications and disfigurement. The remaining half spend the rest of their lives consumed by the constant mental distress of ‘being trapped in the wrong body.’ A vast majority of these individuals suffer from anxiety and depression compounded by hatred, stigmatization, and discrimination on a day-to-day basis.

## Case presentation

A 25-year-old, South Asian assigned-female-at-birth (AFAB) and self-identifying as male with preferred pronouns he/him, presented to us in the outpatient clinic of the psychiatry department with the chief complaints of low mood, suicidal ideations, anxiety, and a desire to change his sex. The fifth of seven siblings, he was born and brought up abroad in an Arab country where his father was a blue-collar worker. As a child, he would dress up as a boy, accompany his father to his workplace, play with cars, and would angrily burn any dolls gifted to him by relatives and friends. He attended a co-educational school up to fifth grade where he got romantically involved with a girl, to the ire of his mother and the school principal. His father was deported to Pakistan when our patient was 12 years old. He and his siblings were stranded abroad for five years with their mother, who had to do odd jobs to make ends meet. He had to drop out of school and developed symptoms of major depression with suicidal ideations during this time. The family finally managed to come back to Pakistan.

His father, who he shared a good relationship with and who would fondly encourage him to dress up as a boy, died two years after the family's arrival in Pakistan. The siblings, along with their mother, now live with an unwelcoming paternal family and do odd jobs to sustain themselves. Our patient, who has always been sexually attracted to women, is currently in a romantic relationship with a female cousin and wants to marry her. They have been kissing but have not yet had sexual intercourse. He practices breast binding and has a strong aversion for his female-specific body parts. He has frequent conflicts with his family because of his lifestyle and suffers from a low mood and suicidal ideations. He denied any specific suicidal plans or intents during his interviews with us. His mother and sisters strongly disapprove of his desire to undergo sex-reassignment while his two younger brothers are supportive of his choice.

During his interviews with us, we found him to be friendly and extroverted, displaying a strong desire to be able to live, dress, and move around like a man. He wants to eventually be able to marry his female cousin, build a home, and have a family. He is a practicing Muslim and reads books on religion besides enjoying poetry, movies, singing, and playing the guitar. He described himself as a pragmatic person who likes to weigh the pros and cons of every decision.

The patient did not have any prior medical or surgical history, including any prior hospital admissions, and did not take any medications. He was born via a normal spontaneous vaginal delivery (SVD) in a hospital and had normal early development. He was playful and energetic as a child and had no neurotic traits or physical illnesses while growing up. He occasionally smokes cigarettes from the age of 14 years. He attained menarche at 14 years of age, has regular periods lasting two to three days with scant blood flow. He was unable to finish his formal high school education after fifth grade and works in a factory for a meager monthly income of a few thousand Pakistani rupees.

On examination, he appeared of his stated age and androgynous, with a slim build. Patchy hair loss was noted in the parietal and occipital regions. The rest of the physical examination was unremarkable. Apart from a dysthymic effect, his mental status examination was unremarkable.

Investigations

The patient was admitted to the inpatient floor of the psychiatry department for medical and psychological evaluation. His vitals were within normal limits. A baseline profile was ordered (including complete blood count (CBC), liver function tests (LFTs), renal function tests (RFTs), and serum electrolytes), which came out to be within normal limits. A gynecology consult was made for a complete gynecological examination, including abdominal and pelvic ultrasound, which was reported to be normal for a female of our patient’s age group. Hormonal levels, including luteinizing hormone (LH), follicle-stimulating hormone (FSH), testosterone, prolactin, and thyroid function tests (TFTs) were also ordered and came out to be within the physiological range for a female of his age group.

Differential diagnosis

Detailed psychological assessment was done to rule out body delusion, transvestism, or female homosexuality.

Treatment

The patient was diagnosed with gender dysphoria with comorbid depression and escitalopram 10 mg by mouth, once daily, was started along with daily psychological therapy. He was advised to express himself according to his desired gender during these sessions and was also educated about the standard requirement of having to live as a person of the desired gender in the society for at least six months before any sex reassignment procedures could be initiated. The possible societal, emotional, and legal challenges associated with sex reassignment surgeries in Pakistan were described in detail. Endocrinology consult was called to comment about future hormonal therapy options, but they refused to comment fearing the legal repercussions of any such intervention.

Outcome and follow-up

During his two week stay in the hospital, our patient was under immense pressure from his family, especially his mother who was vehemently opposed to any form of sex-reassignment procedures for religious reasons. After a fortnight of medical and psychological therapy, he was sent home and advised to follow up in the outpatient clinic weekly for the next six months while continuing to live as a man at home and at work. He, however, subsequently failed to follow up with us, citing clashing work hours, familial resistance, and a lack of financial independence as the main reasons.

## Discussion

The reporting of this case is of utmost significance because, on a global level, transgender health remains a neglected and under-studied subject [15]. It will be the first case of female-to-male gender dysphoria from Pakistan to be reported in the literature. To date, only two cases of male-to-female gender dysphoria (previously gender identity disorder) from Pakistan have been reported [16-17].

The reporting of this case is also significant because no effort has been made in earlier case reports on gender dysphoria from Pakistan to acknowledge or use the patients’ preferred pronouns. Given the increasing global awareness around the proper use of preferred pronouns, we have taken great care to always use the patient's preferred pronouns and gender identity throughout this case report.

This report is also significant because it will also be the first case report on this subject from Pakistan after the DSM-5's recoining of the previously used term gender identity disorder as gender dysphoria (to better emphasize the fact that it is the distress from being trapped in the wrong body that is pathological and not the feeling itself).

Socioeconomic factors are very important determinants of the final outcome in gender dysphoria patients, and existing case reports from Pakistan do not make any reference to the socioeconomic status of their patients. Therefore, it is imperative to report this case because it details the impact of socioeconomic determinants on the final outcomes in gender dysphoria patients in low-income, Muslim, Pakistani households.

## Conclusions

It is imperative to expose medical students to transgender individuals during the course of their education and to teach them about the unique physical and mental health needs of transgendered bodies. At the same time, serious discourse must also begin among Pakistani medical and legal experts, as well as human rights activists, about court requisitions for sex-reassignment surgeries and whether such a prerequisite negates the fundamental right of a capable, adult human being to be able to make decisions about their own body. There is a dire need for a wide-scale social awareness campaign on national television and print and social media in order to educate the general public about the discrimination, hatred, and violence traditionally faced by transgender individuals. At the same time, parents of transgender children must be educated about the importance of providing their children unconditional love and emotional support to help them grow into strong, independent individuals.

## References

[1] American Psychiatric Association (2013). Diagnostic and Statistical Manual of Mental Disorders (DSM-5®), Fifth Edition. https://www.appi.org/Diagnostic_and_Statistical_Manual_of_Mental_Disorders_DSM-5_Fifth_Edition.

[2] (2020). Population census. http://www.pbs.gov.pk/content/population-census.

[3] Jami H, Kamal A (2015). Measuring attitudes toward hijras in Pakistan: gender and religiosity in perspective. Pak J Psychol Res.

[4] Alizai A, Doneys P, Doane D (2017). Impact of gender binarism on hijras’ life course and their access to fundamental human rights in Pakistan. J Homosex.

[5] Khan F (2020). Khwaja sira: culture, identity politics, and "transgender" activism in Pakistan. Syracuse University.

[6] Azhar M (2020). Pakistan's traditional third gender isn't happy with the trans movement. http://www.pri.org/stories/2017-07-29/pakistans-traditional-third-gender-isnt-happy-trans-movement.

[7] Ingber S (2020). Pakistan passes historic transgender rights bill. http://www.npr.org/sections/thetwo-way/2018/05/09/609700652/pakistan-passes-historic-transgender-rights-bill.

[8] Ming L, Hadi M, Khan T (2016). Transgender health in India and Pakistan. Lancet.

[9] Redding J (2015). From 'she-males' to 'unix': transgender rights and the productive paradoxes of Pakistani policing. Regimes of Legality: Ethnography of Criminal Cases in South Asia.

[10] Phullan B (2020). Violence against transgender. http://nation.com.pk/27-Aug-2018/violence-against-transgender.

[11] Smith R (2020). Pakistani transgender woman 'kidnapped and tortured' by gang. http://www.pinknews.co.uk/2019/06/11/pakistani-transgender-woman-kidnapped/.

[12] Khan A, Shekhani S (2020). Are doctors ready for the medical needs of the transgender community?. https://www.dawn.com/news/1472414.

[13] Imran M (2020). Person diagnosed with gender dysphoria seeks IHC permission for sex reassignment. http://www.dawn.com/news/1393533.

[14] Gale J (2020). How Thailand became a global gender-change destination. https://www.bloomberg.com/news/features/2015-10-26/how-thailand-became-a-global-gender-change-destination.

[15] Reisner S, Poteat T, Keately J (2016). Global health burden and needs of transgender populations: a review. Lancet.

[16] Yousafzai A, Bhutto N (2017). Gender identity disorder. Is this a potentially fatal condition?. J Ayub Med Coll Abbottabad.

[17] Abidi MA, Ullah H (2001). Gender identity disorder. J Coll Physicians Surg Pak.

